# Advancing global sea ice prediction capabilities using a fully coupled climate model with integrated machine learning

**DOI:** 10.1126/sciadv.ady8957

**Published:** 2026-01-01

**Authors:** William Gregory, Mitchell Bushuk, Yong-Fei Zhang, Alistair Adcroft, Laure Zanna, Colleen McHugh, Liwei Jia

**Affiliations:** ^1^ Atmospheric and Oceanic Sciences Program, Princeton University, Princeton, NJ 08540, USA.; ^2^ NOAA, Geophysical Fluid Dynamics Laboratory, Princeton, NJ 08540, USA.; ^3^ Earth System Science Interdisciplinary Center, University of Maryland, College Park, MD 20740, USA.; ^4^ Courant Institute of Mathematical Sciences, New York University, New York, NY 10012, USA.; ^5^ Center for Data Science, New York University, New York, NY 10011, USA.; ^6^ Science Applications International Corporation, Reston, VA 20190, USA.

## Abstract

We showcase a hybrid modeling framework that embeds machine learning (ML) inference into the Geophysical Fluid Dynamics Laboratory Seamless System for Prediction and Earth System Research (SPEAR) climate model for online sea ice bias correction during a set of global fully coupled 1-year retrospective forecasts. We compare two hybrid versions of SPEAR to understand the importance of exposing ML models to coupled ice-atmosphere-ocean feedbacks before implementation into fully coupled simulations: Hybrid_CPL_ (couple trained; with feedbacks) and Hybrid_IO_ (ice ocean trained; without feedbacks). Relative to SPEAR, Hybrid_CPL_ systematically reduces seasonal forecast errors in the Arctic and considerably reduces Antarctic errors for target months May to December, with >2× error reduction in 4- to 6-month lead forecasts of Antarctic winter sea ice extent. Meanwhile, Hybrid_IO_ suffers from out-of-sample behavior that can trigger a chain of Southern Ocean feedbacks, leading to ice-free Antarctic summers. Our results emphasize that ML can demonstrably improve numerical sea ice prediction capabilities and that exposing ML models to coupled ice-atmosphere-ocean processes is essential for generalization in fully coupled simulations.

## Teaser

Machine learning models must be exposed to coupled climate processes to generalize in future climate model predictions.

## INTRODUCTION

Over the past 4 to 5 decades, remote sensing observations, ground-based instruments, and submarine surveys have shown that Earth’s sea ice cover is undergoing marked changes. The Arctic, for example, has seen basin-wide thinning and retreat of sea ice across all seasons (*1*, *2*). This ice loss has played a significant role in high-latitude climate feedbacks and Arctic amplification, where Arctic surface temperatures have warmed at nearly four times the rate of the global average (*3*). Furthermore, Arctic sea ice loss can also contribute to a slowdown in the poleward transport of warm ocean waters (*4*) and increased frequency of extreme weather events across Europe (*5*) and North America (*6*, *7*). Meanwhile, Antarctic sea ice area exhibited a modest positive trend between 1979 and 2014. However, since 2016, there have been five record low summer minima and two record low winter maxima, with many studies now suggesting a regime shift in Antarctic sea ice caused by Southern Ocean warming (*8*–*12*).

Reproducing historical sea ice changes within climate models is critical for enabling confident assessments of how both anthropogenic forcing and internal climate variability will shape future sea ice evolution and its impacts on climate and society. Meanwhile, the latest generation of climate models submitted to the sixth phase of the Coupled Model Intercomparison Project (CMIP6) shows considerable spread in their simulations of historical sea ice area and trends, with models generally underestimating the sensitivity of sea ice to global warming in the Arctic (*13*) and overestimating the sensitivity in the Antarctic (*14*). While internal climate variability certainly contributes to this spread (*15*, *16*), errors in component and coupled model physics remain the dominant source of historical bias and mid–21st century projection uncertainty (*17*).

On shorter timescales, these model physics errors also affect our ability to make accurate seasonal-to-interannual sea ice predictions, as models struggle to faithfully reproduce various physical drivers of regional sea ice variability (*18*–*20*). Since 2008, there has been a growing community effort to understand and improve sea ice prediction capabilities. This effort culminates each year into a “Sea Ice Outlook,” where community members submit seasonal forecasts of the September Arctic sea ice minimum and February Antarctic sea ice minimum to the Sea Ice Prediction Network (SIPN) online platform (*21*, *22*). Forecasts range from statistical techniques (*23*–*25*) to fully coupled dynamical models (*26*–*28*), as well as heuristic approaches. A recent intercomparison of 34 individual forecast systems that are routinely submitted to SIPN found that many statistical and dynamical models can skillfully predict September Arctic sea ice conditions 1 to 3 months in advance (*29*), suggesting that useful real-time predictions of September Arctic sea ice are likely on the horizon. Meanwhile, in a separate SIPN South intercomparison study of Antarctic forecasts, statistical models were found to generally outperform coupled climate models at predicting regional-scale sea ice variability (*22*). This therefore prompts an urgent need to improve Antarctic sea ice forecasts within climate models.

Achieving useful seasonal-to-interannual climate model sea ice predictions means addressing both the model physics errors that lead to systematic bias and also ensuring accurate initial conditions for the land, atmosphere, ocean, and sea ice. Accurate initial conditions are routinely achieved through frameworks such as nudging (*30*–*32*) and data assimilation (DA) (*33*–*35*). Within which, model states are either linearly relaxed toward a set of observations over a given time window (nudging) or updated through a Bayesian treatment of model and observational uncertainty (DA). In this present study, we investigate specifically the model physics problem, while also using DA to characterize model errors.

The recent growth in application of machine learning (ML) techniques to climate research has been extraordinary. For sea ice, this has led to breakthroughs in remote sensing, sea ice reanalysis (*36*–*39*), and statistical forecasting (*40*, *41*) and has also paved the way for an era in “hybrid” sea ice modeling, using ML to replace or improve certain aspects of sea ice model physics (*42*–*45*). Of course, hybrid modeling is not only limited to sea ice but has also been a burgeoning area of research in both atmosphere (*46*–*49*) and ocean (*50*–*53*) models. One branch of hybrid climate modeling in particular focuses on learning state-dependent representations of structural model error. In this approach, it is assumed that the corrections, or increments, applied to a numerical simulation during DA or nudging are largely a manifestation of predictable errors associated with poorly parameterized/missing physics and the discretization of continuous equations (*54*). An ML model can therefore be used to predict these increments using only model state variables as inputs, thus providing a framework for online bias correction during subsequent numerical simulations. This approach has been shown to successfully reduce systematic model biases when run in component and idealized models (*45*, *48*, *55*, *56*). A recent study also extended this approach to bias correct sea ice and ocean conditions in the fully coupled Norwegian Climate Prediction Model (*57*). While their study showed promising bias improvements, their implementation was restricted to the Arctic domain and used different ML models for each prediction month and year, resulting in 236 different ML models. Their approach also only performed bias correction once per roughly 15 days.

In this present article, we seek to understand the importance of exposing ML models to coupled ice-atmosphere-ocean climate feedbacks before their implementation into fully coupled numerical simulations. To do this, we create two hybrid versions of the Geophysical Fluid Dynamics Laboratory (GFDL) seasonal-to-decadal prediction model, Seamless System for Prediction and Earth System Research (SPEAR) (*58*). The first hybrid model, Hybrid_IO_, has an ML component that is trained to predict sea ice concentration (SIC) error corrections from a reanalysis-forced ice-ocean (IO) configuration of SPEAR, which performs SIC DA and sea surface temperature (SST) nudging. The second hybrid model, Hybrid_CPL_, has an ML component that is trained to predict SIC error corrections from a fully coupled configuration of SPEAR, which performs SIC DA and nudges SST and the three-dimensional (3D) atmosphere, temperature, wind, and humidity fields. The fundamental difference between Hybrid_IO_ and Hybrid_CPL_ is that the training data for Hybrid_CPL_ were generated from a simulation that allows for coupled ice-atmosphere-ocean feedbacks, while the training simulation for Hybrid_IO_ does not. This will therefore allow us to determine the importance of these feedbacks when training ML models for implementation into free-running (no nudging) fully coupled simulations. In this study, we compare the hybrid models in a suite of global, 1-year, fully coupled retrospective forecast (reforecast) experiments, initialized over a 6-year period between 2018 and 2023.

Previous studies have provided seminal work on how offline-trained ML models can suffer generalization issues when implemented into online numerical simulations due to feedbacks between the ML parameterization and the dynamical model, which the ML model did not see during training (*59*, *60*). Our present work is distinct from these studies in two key ways: (i) We address the issue of offline-to-online generalization from the outset by fine-tuning all ML models using a data augmentation procedure, which iteratively exposes the ML model to online feedbacks (*45*) (see Materials and Methods). This allows us to focus specifically on the impacts of ice-atmosphere-ocean climate feedbacks on ML generalization. (ii) While past studies tested offline-to-online generalization in a single-component idealized (Lorenz-96) system, we conduct our analysis in global fully coupled simulations using SPEAR.

The paper proceeds as follows: Results first evaluates the year-round forecast skill of both Hybrid_IO_ and Hybrid_CPL_, relative to satellite observations of SIC from the National Snow and Ice Data Center (NSIDC) NASA Team dataset (*61*). We then investigate why Hybrid_IO_ systematically degrades forecast performance in Arctic and Antarctic summer, paying attention to generalization of network inputs and evaluation of coupled climate feedbacks. We show that, through including coupled feedbacks in the training data, Hybrid_CPL_ simulations are better able to generalize to free-running coupled simulations. Last, we explore the potential for Hybrid_CPL_ to predict extreme events and discuss its future outlook for climate-timescale integrations.

## RESULTS

The 1-year reforecasts in this study are based on 15-member ensemble predictions that are initialized on the first day of each month for all months between January 2018 and December 2023. This provides 72 reforecasts to evaluate in the Arctic and Antarctic. All results are based on the ensemble mean of all 15 members (see Materials and Methods for a description of the ensemble).

### Performance of hybrid model forecasts

**Fig. 1.**
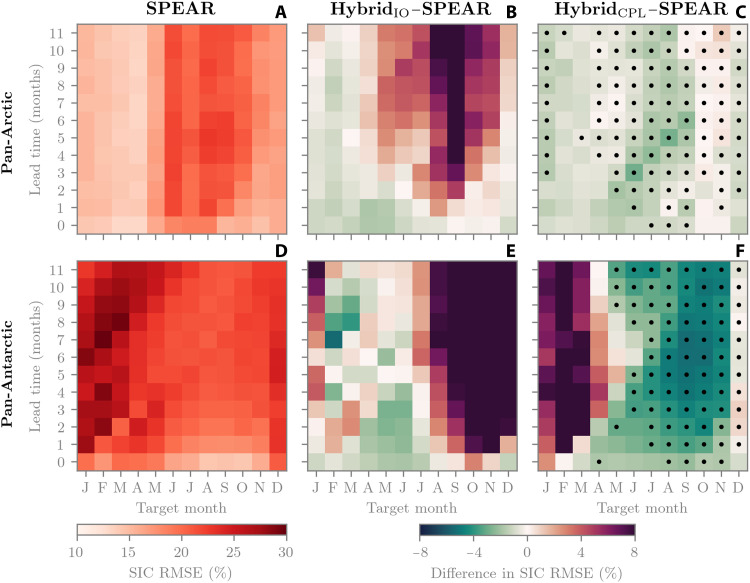
SIC prediction error, 2018–2024. (**A**) SPEAR pan-Arctic RMSE. (**B**) RMSE difference between Hybrid_IO_ and SPEAR. (**C**) Same as (B) but for Hybrid_CPL_. (**D** to **F**) Same as for (A) to (C) but for pan-Antarctic. Stippling in (C) and (F) shows where Hybrid_CPL_ outperforms Hybrid_IO_ at the 95% confidence level. RMSE is computed relative to NSIDC observations.

Figure 1 shows the root mean square error (RMSE) of Arctic and Antarctic SIC predictions for each target and initialization month. Here, the RMSE corresponding to a lead 0 prediction of January is computed as the mean of daily RMSEs between 1 and 31 January from a 1 January–initialized forecast. A lead 1 prediction of January is then the mean of daily RMSEs between 1 and 31 January from a 1 December–initialized forecast and so on. Daily RMSEs are evaluated over grid points where SIC is greater than zero in either the observations or model. For SPEAR (Fig. 1, A and D), the RMSE is highest for summer target months in both hemispheres, although the Antarctic generally displays higher year-round RMSE. Larger summertime errors are expected, given that ice melting causes local SIC variations throughout the interior ice pack, while, in winter, the interior ice pack is predominantly fully ice covered in both observations and models. Figure 1 (B and E) then shows the difference in RMSE between Hybrid_IO_ and SPEAR, where green colors indicate an improved forecast relative to SPEAR and red colors indicate a poorer forecast. The degradation in May to November Arctic predictions and July to January Antarctic predictions is the most notable features of these panels. Meanwhile, Arctic predictions in Hybrid_CPL_ are near systematically improved compared to SPEAR (Fig. 1C) and are significantly improved (95% confidence, estimated by a 10,000 sample bootstrapping with replacement) in 72% of cases over Hybrid_IO_. Between May and December, Hybrid_CPL_ systematically reduces Antarctic RMSE relative to SPEAR and Hybrid_IO_. However, forecasts are degraded between January and April (discussed in detail in a later section on coupled model biases). Overall, Hybrid_CPL_ shows improvement over Hybrid_IO_ in 56% of cases in the Antarctic, although, with only 7 years of validation, these values could be subject to internal variability.

**Fig. 2.**
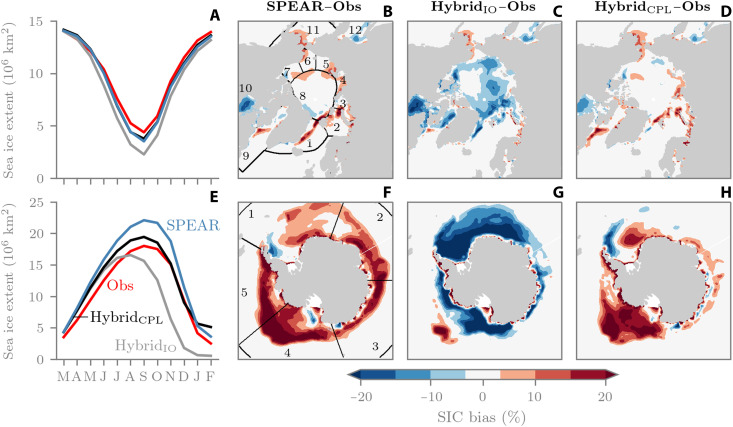
March-initialized reforecast bias, 2018–2024. (**A**) Mean pan-Arctic sea ice extent. (**B** to **D**) SIC bias across entire 1-year reforecasts for SPEAR, Hybrid_IO_, and Hybrid_CPL_, respectively. (**E** to **H**) Same as (A) to (D) but for Antarctic. Biases are relative to NSIDC observations (Obs). Regions in (B) are as follows: 1, GIN Sea; 2, Barents Sea; 3, Kara Sea; 4, Laptev Sea; 5, East Siberian Sea; 6, Chukchi Sea; 7, Beaufort Sea; 8, Central Arctic; 9, Baffin Bay and Labrador Sea; 10, Hudson Bay; 11, Bering Sea; 12, Sea of Okhotsk. Regions in (F) are as follows: 1, Weddell Sea; 2, Indian Ocean; 3, Pacific Ocean; 4, Ross Sea; 5, Amundsen and Bellingshausen Sea.

To take a closer look at the performance of each model, Fig. 2 now shows March-initialized reforecast biases. Starting with the Arctic, the baseline SPEAR model generally performs well at Arctic sea ice forecasts, ranking second against 16 other dynamical model predictions of September Arctic sea ice in a recent intercomparison (*29*). In Fig. 2A, we can see that SPEAR (blue line) tracks the observed pan-Arctic extent (red line) well from March to June, although it starts to diverge in July and slightly underpredicts the September minimum. The mean SIC error across the 1-year reforecasts (Fig. 2B) then shows that SPEAR has too much sea ice in places such as the Greenland, Iceland, and Norwegian (GIN), Barents, Laptev, Chukchi, and Bering seas, and too little sea ice in Hudson Bay and the Sea of Okhotsk. Figure S1 shows a breakdown of the SPEAR reforecast biases month by month, highlighting that the summertime low bias originates in Hudson Bay and Baffin Bay in June and then spreads to the Canadian Archipelago by August and September. Meanwhile, the Hybrid_IO_ sea ice extent (gray line) starts to diverge from observations in May (Fig. 2A), resulting in systematic underprediction for the remainder of the forecast period. Figure 2C then shows that Hybrid_IO_ has overcorrected the majority of SPEAR’s positive SIC biases, with now predominantly negative SIC biases relative to observations (in the next section, we attribute this bias reversal to out-of-sample ML inputs). For Hybrid_CPL_, pan-Arctic extent is largely overlapping with SPEAR (Fig. 2A, black line) but displays improved performance for local SIC predictions (Fig. 2D). Some noteworthy features include a near eradication of a systematic GIN Sea bias, along with improvements in Hudson Bay, the East Siberian Sea, the Beaufort Sea, and the Sea of Okhotsk.

Turning to the Antarctic (Fig. 2E), SPEAR has a systematic circumpolar, year-round positive sea ice extent bias, which is largest in austral winter and has particularly significant contributions from places such as the Ross, Amundsen, and Bellingshausen seas (Fig. 2F). For Hybrid_IO_, pan-Antarctic sea ice extent reaches its maximum in August, a full month earlier than both observations and SPEAR (Fig. 2E). From this point on, the sea ice extent declines until reaching ice-free conditions (less than 1 million km^2^ of extent) by February, exemplified by the near hemisphere-wide negative SIC bias in Fig. 2G; we explain that coupled feedbacks drive this pathological behavior in the next section. For Hybrid_CPL_, pan-Antarctic extent is improved relative to SPEAR in all months except February (Fig. 2E). The positive extent bias in winter is markedly reduced, and the early melt season extent (October to December) tracks the observations very well. Figure 2H highlights notable bias improvements in the Indian and Pacific sectors, the Weddell Sea, and the Amundsen and Bellingshausen seas. However, degradations exist in the southern Ross Sea (discussed later).

### Diagnosing forecast discrepancies and the role of coupled climate feedbacks

**Fig. 3.**
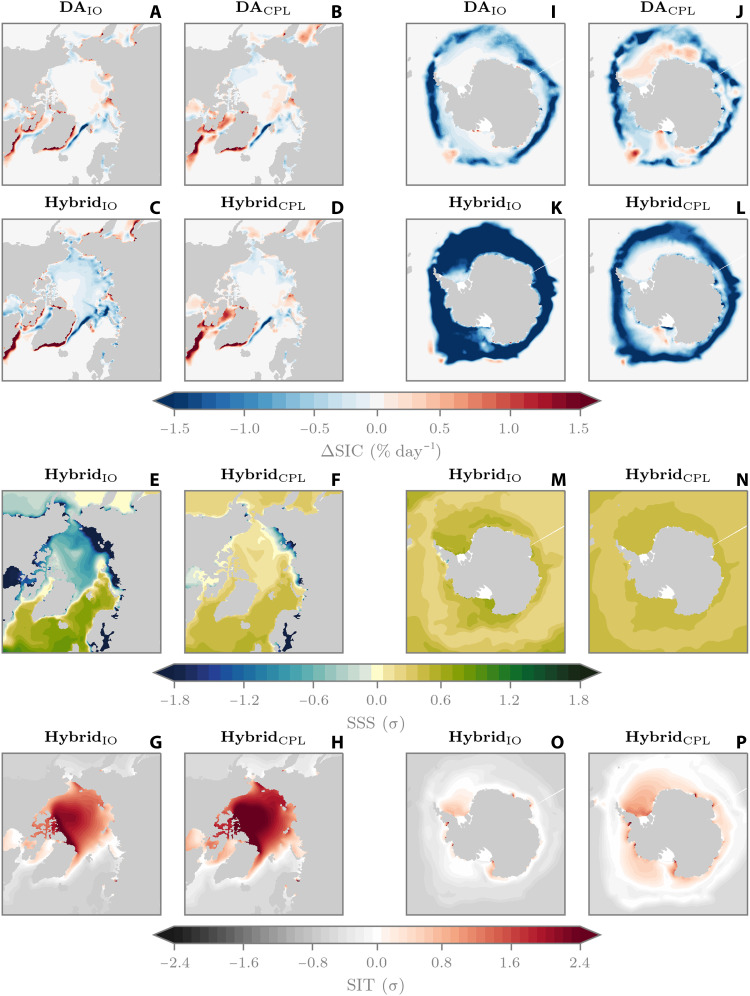
Ice-ocean and coupled SIC increments and ML inputs. (**A**) Mean March to July SIC increments from DA between 1982 and 2017 from the reanalysis-forced ice-ocean (IO) simulation. (**B**) Same as (A) but for the coupled (CPL) simulation with atmospheric nudging. (**C** and **D**) Mean 2018–2024 March to July ML increments from March-initialized reforecasts with Hybrid_IO_ and Hybrid_CPL_, respectively. (**E** and **F**) Same as (C) and (D) but for normalized SSS. (**G** and **H**) Same as (E) and (F) but for normalized sea ice thickness (SIT). (**I** to **P**) Same as (A) to (H) but for Antarctic June to August period.

To understand why Hybrid_IO_ systematically underpredicts sea ice conditions in the Arctic and produces ice-free Antarctic summers, we first investigate potential out-of-sample issues. Starting with the Arctic, we look at the March to July period where the March-initialized Hybrid_IO_ reforecasts start to diverge from SPEAR and Hybrid_CPL_. Figure 3 (A and B) shows the mean March to July SIC increments from the 36-year ice-ocean DA simulation and the fully coupled DA simulation, respectively. Here, we can see that the increments show overall very similar magnitudes and spatial patterns, highlighting that the ice-ocean and coupled models have similar sea ice biases in the Arctic. When we then look at the March-initialized reforecasts, we can see that the ML increments from Hybrid_IO_ are generally negative within the Arctic basin and have larger magnitudes than the ice-ocean DA experiment (Fig. 3, C versus A). Meanwhile, the increments from Hybrid_CPL_ are in good agreement with the nudged DA experiment (Fig. 3, D versus B). Diagnosing each of the inputs to the ML models reveals that sea surface salinity (SSS) may be causing an out-of-sample issue for Hybrid_IO_ (Fig. 3, E and F). This is because the ice-ocean DA experiment also includes a restoring of SSS to a monthly climatology, whereas the SSS is allowed to evolve freely in the coupled DA experiment. Therefore, normalizing SSS during Hybrid_IO_ reforecasts based on the statistics of the ice-ocean DA experiment produces SSS values of >4σ lower than Hybrid_CPL_ in places such as Hudson Bay and the Eurasian coastal seas and ~0.5σ lower across the Arctic basin. This highlights that SPEAR generally has a fresher ocean surface than the SSS-restored ice-ocean model. This out-of-sample behavior also explains why, relative to SPEAR, Hybrid_IO_’s SIC bias pattern appears to flip sign in the Arctic basin (Fig. 2, C versus B). Both the coupled model and ice-ocean model have a positive SIC bias within the Arctic basin; hence, Hybrid_IO_ and Hybrid_CPL_ predict negative SIC increments in this region on average. However, because of out-of-sample network inputs, the increments from Hybrid_IO_ become too large, which overcorrects the positive reforecast bias and reverses the sign.

Conducting the same analysis between June and August for the Antarctic reveals different increment spatial patterns between the two DA experiments (Fig. 3, I and J). These differences are most notable in the Weddell Sea, where the ice-ocean DA increments are slightly negative within the interior ice pack, while the coupled DA increments are positive; this emphasizes different sea ice biases between the ice-ocean and coupled models. The Hybrid_IO_ increments are then systematically negative in the Weddell Sea and are over 2× larger in magnitude than those from DA (compare Fig. 3, K and I). Meanwhile, the increments from Hybrid_CPL_ are more in line with those from the coupled DA experiment, although they show lower-magnitude positive increments in the Weddell and Ross seas and larger-magnitude negative increments in the marginal ice zone (Fig. 3, L versus J). This time, the normalized SSS fields between Hybrid_IO_ and Hybrid_CPL_ are very similar and generally “in sample” (Fig. 3, M and N), and the largest differences in ML inputs occur in sea ice thickness (SIT; Fig. 3, O versus P). Between June and August, the mean pan-Antarctic sea ice extent in Hybrid_IO_ is ~7% lower than the respective Hybrid_CPL_ mean extent. However, the mean pan-Antarctic SIT in Hybrid_IO_ is over 25% lower than Hybrid_CPL_. In the Arctic, Hybrid_IO_ is 3% thicker than Hybrid_CPL_ on average. However, the normalized Hybrid_IO_ SIT values are slightly lower in magnitude than Hybrid_CPL_ (Fig. 3, G versus H). This is primarily being influenced by the fact that the nudged coupled DA simulation has lower SIT variability than the ice-ocean DA run. Therefore, normalizing Hybrid_CPL_ reforecasts by a smaller SD produces larger normalized SIT.

**Fig. 4.**
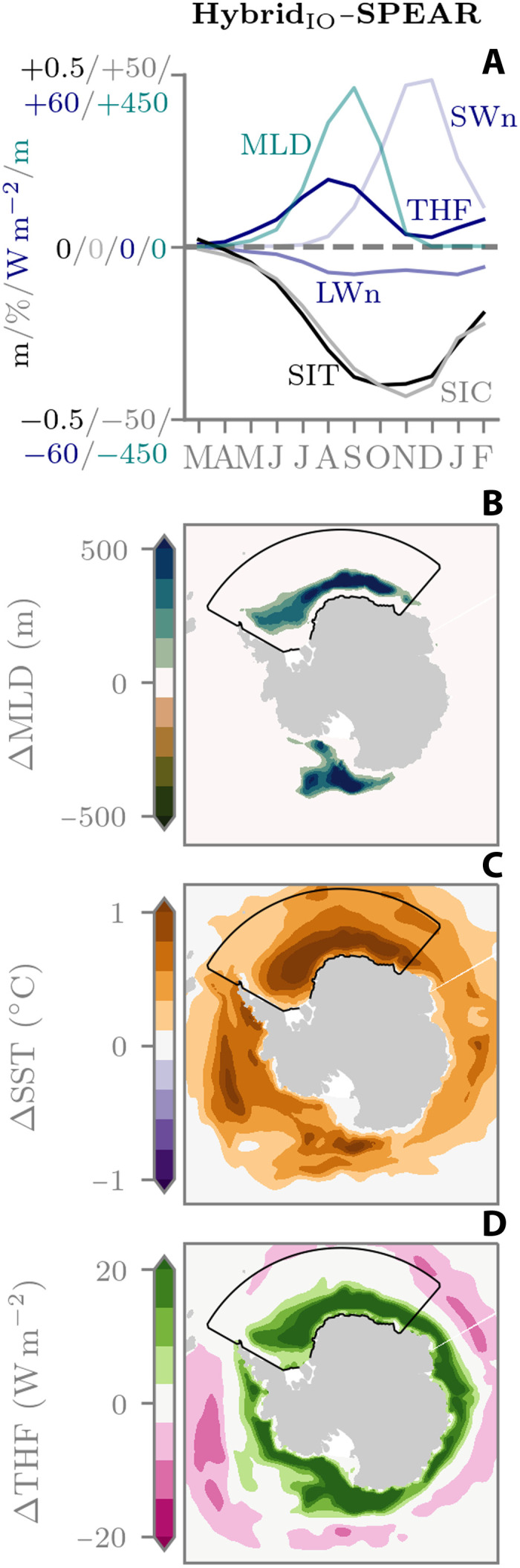
March-initialized Hybrid_IO_-SPEAR anomalies, 2018–2024. (**A**) Mean Weddell Sea anomalies in SIC, SIT, SWn radiation, LWn radiation, THF, and MLD. THF sign convention is positive upward, while LW and SW are positive downward. (**B** to **D**). Average Hybrid_IO_ anomalies in MLD, SST, and THF across the 1-year reforecasts. Contour shows region of anomalies in (A).

At this point it is worth noting that the ML models in this study do not predict SIT increments but rather make changes to the model’s SIT by adjusting the concentration of ice within each of the model’s ice thickness categories (see Materials and Methods). A question therefore remains as to whether Hybrid_IO_’s thinner and less extensive ice between June and August is coming directly from the ML model’s SIC updates or whether the ML model is also triggering feedbacks that inhibit winter ice growth rates and ultimately lead to ice-free conditions by the end of summer. To investigate this further, we take a process-oriented approach by looking at anomalies in coupled ice-atmosphere-ocean diagnostics relative to SPEAR. Figure 4A shows mean Weddell Sea (48.5°W to 39.5°E, 56.61°S to 90°S) anomalies in SIC, SIT, mixed-layer depth (MLD), and surface energy balance terms for each month of the March-initialized reforecasts. Note that the surface energy balance corresponds to the sum of net shortwave (SWn), net longwave (LWn), and turbulent heat fluxes (THFs), where THFs are the sum of sensible and latent heat exchanges. THFs are also defined as positive upward, while LWn and SWn are positive downward. For this region of the Weddell Sea, the negative SIC and SIT anomalies indicate an overall negative sea ice volume anomaly relative to SPEAR between March and August. This sea ice volume anomaly is accompanied by a deepening of the ocean mixed layer (~300 m), as well as an increase in both THF (~20 W m^−2^) and upward longwave (~10 W m^−2^). This can be explained by the volume anomaly creating areas of open water and thinning the sea ice, both of which make the ocean more susceptible to surface forcing from the atmosphere. This cold wintertime forcing then drives surface cooling and ocean convection (Fig. 4B), which brings relatively warm and saline waters to the surface (Fig. 4C) and increases THF (Fig. 4D), all of which inhibit winter ice growth rates. Between March and August solar insolation is also at its lowest, resulting in little to no response from SWn. However, by the time shortwave “turns on” in September, the volume anomaly has already had a marked impact on the surface albedo. A positive SWn anomaly then grows between September and November and coincides with higher rates of ice loss in Hybrid_IO_ (compare Hybrid_IO_ and SPEAR sea ice extent curves in Fig. 2E). This indicates that the wintertime ocean preconditioning of the sea ice is also potentially triggering summertime ice-albedo feedbacks, further enhancing the sea ice anomaly. The reason that the SIC and SIT anomalies in Fig. 4A then start to recover between December and February is because Hybrid_IO_ has effectively lost its ice cover.

Antarctic reforecasts with Hybrid_IO_ appear to be an example of how interactions between ML models and climate physics can cause out-of-sample behavior and potential runaway feedbacks. This occurs by the ML model preconditioning the winter sea ice and ocean state to facilitate ice-free conditions by end of summer. Evaluating the same Fig. 4 diagnostics for Hybrid_CPL_ reveals a stable simulation with no sizable anomalies relative to SPEAR (fig. S2). This highlights that ice-atmosphere-ocean feedbacks within ML training data are essential for online generalization in coupled models, in this case, by preventing a chain of coupled feedbacks between the sea ice, ocean interior, and the surface atmosphere. Last, evaluating Hybrid_IO_ in the Arctic also does not show the same pathological behavior as the Antarctic, with mean anomalies in surface energy balance terms on the order of 1 W m^−2^ and MLD anomalies of less than 5 m across the Arctic basin (see fig. S3). This may indicate that an ML model trained in an ice-ocean configuration could generalize to the fully coupled SPEAR model in the Arctic after careful treatment of nudging routines, such as SSS. We note here that the Arctic is generally better behaved than the Antarctic in both Hybrid_IO_ and Hybrid_CPL_ (recall Fig. 1). We can attribute this to the fact that, in both SPEAR and observations, the Arctic Ocean is much more stratified than the Southern Ocean (see fig. S4). This means that Arctic sea ice is more isolated from the ocean interior and thus has less potential to be influenced by interior ocean model errors. Meanwhile, a well-mixed Southern Ocean means that ocean processes (and biases) are more tightly coupled to the sea ice and have the potential to cause generalization issues for hybrid sea ice models, as we show in the next section.

### Impact of coupled model biases on ML generalization

At this point, we established that Hybrid_CPL_ is the desirable hybrid model for global sea ice bias correction through its ability to generalize to online ice-atmosphere-ocean climate feedbacks. However, in Fig. 1F, we saw that Hybrid_CPL_ also shows degradations in forecast skill relative to SPEAR for target months in Antarctic summer (January to April). Given that these biases occur during the melt and early growth season, it is reasonable to expect that Hybrid_CPL_ could be inadequately capturing melt and growth processes. However, a mass budget decomposition (see fig. S5) reveals that both the thermodynamic and dynamic terms contributing to sea ice mass evolution are very similar between SPEAR and Hybrid_CPL_, suggesting feasible melt and growth processes in Hybrid_CPL_. Instead, we show in this section that these summertime degradations originate from an out-of-sample problem related to coupled model biases.

**Fig. 5.**
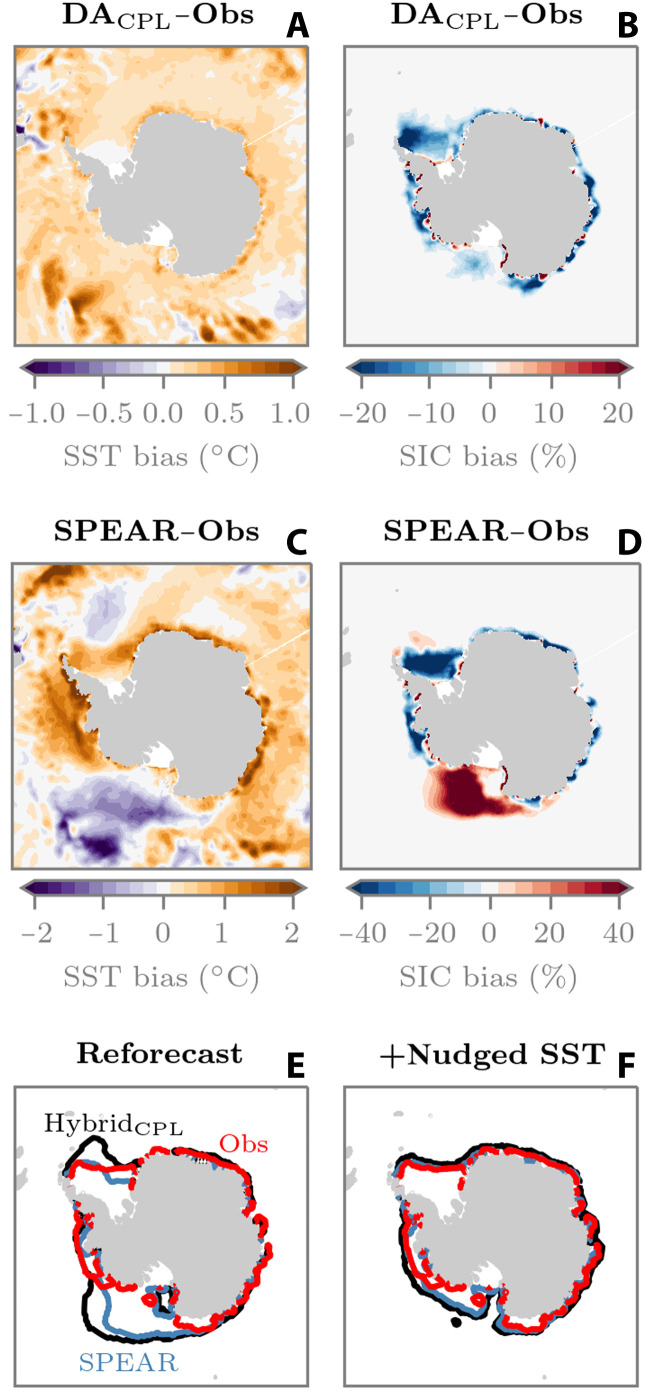
February-mean Antarctic sea ice and ocean biases. (**A** and **B**) Thirty-six–year (1982–2017) SST and SIC biases from the coupled DA experiment, respectively. SST bias relative to Optimum Interpolation SST (OISST) data (*76*) and SIC bias relative to NSIDC observations. (**C** and **D**) Same as (A) and (B) but for November-initialized SPEAR reforecasts of February, 2018–2024. (**E** and **F**) February sea ice edge locations from November-initialized reforecasts without and with SST nudging, respectively.

We recalled that the Hybrid_CPL_ ML model was trained on model state variables that were generated from a simulation, which performs SIC DA as well as SST and atmospheric nudging. We then implemented this ML model into reforecast experiments with a free-running atmosphere and ocean. Learning DA increments in this nudged configuration was intended to create an environment in which the ML model learns intrinsic sea ice model physics errors, as opposed to coupled model biases, which imprint on the sea ice. However, if the ML model has not been exposed to these biases, then it could make erroneous online predictions. In Fig. 5A, we can see that the 36-year coupled DA simulation (which performs SST nudging) contains a slight positive summertime (February) SST bias. The resultant February SIC from this simulation (Fig. 5B) also has an Antarctic-wide low bias; the February SIC DA increments will therefore be positive to counteract this bias. In the free-running coupled reforecasts, SPEAR exhibits larger and more heterogeneous February SST biases (Fig. 5C; we use November-initialized forecasts of February as an example here, but the same relationship holds for other initialization dates and summer target months). One noteworthy region is the Ross Sea, which contains a large area of negative (cold) SST bias. In Fig. 5D, we then see this SST bias imprinted onto the sea ice as a positive SIC bias. On the basis of the DA simulation, the ML model has learned to add sea ice in Antarctic summer. However, in the online reforecasts, it is now adding sea ice onto a preexisting positive Ross Sea bias. We can see this in Fig. 5E, which shows the February sea ice edge contour for each reforecast experiment, highlighting the fact that Hybrid_CPL_ has exacerbated the sea ice bias in the Ross Sea. Furthermore, Hybrid_CPL_ has also exacerbated a slight positive bias in the Weddell Sea, which may also be related to the negative SST bias in this location (see Fig. 5C). This positive sea ice extent bias is also reflected in the sea ice mass budget terms (fig. S5), where a more extensive sea ice cover means an increase in sea ice mass loss due to bottom melt processes. We further test our hypothesis of an ocean-related generalization problem by repeating the November-initialized reforecasts, but this time with SST nudging turned on. In Fig. 5F, we can see that Hybrid_CPL_ performs better in this scenario, with a sea ice edge that is in closer agreement with observations and SPEAR in the Ross and Weddell seas.

These results support the notion that, while Hybrid_CPL_ is better equipped to handle the coupled climate feedbacks that affect sea ice evolution, the well-mixed Southern Ocean (recall fig. S4) means that the ML model is still susceptible to out-of-sample behavior through interactions with coupled model biases. It is therefore reasonable to assume that if these Southern Ocean biases were addressed, then Hybrid_CPL_ would likely yield systematic year-round forecast improvements in both the Arctic and Antarctic; we outline potential future directions to this end in Discussion.

### Extreme events: September 2023 Antarctic case study

We now learn that coupled feedbacks play a central role in Hybrid_CPL_’s ability to, on average, improve seasonal sea ice forecast skill. We therefore conclude Results with a case study to determine the potential for Hybrid_CPL_ to also yield improved forecasts in extreme years. The 2023 September Antarctic sea ice extent gained considerable attention for being a “once in a multimillion-year event” with an extent anomaly >5σ below the 1980–2010 mean (*62*). This anomaly was primarily caused by anomalously warm upper ocean temperatures and strong northerly winds, both of which significantly inhibited winter ice growth rates in the Ross and Weddell seas (*63*).

**Fig. 6.**
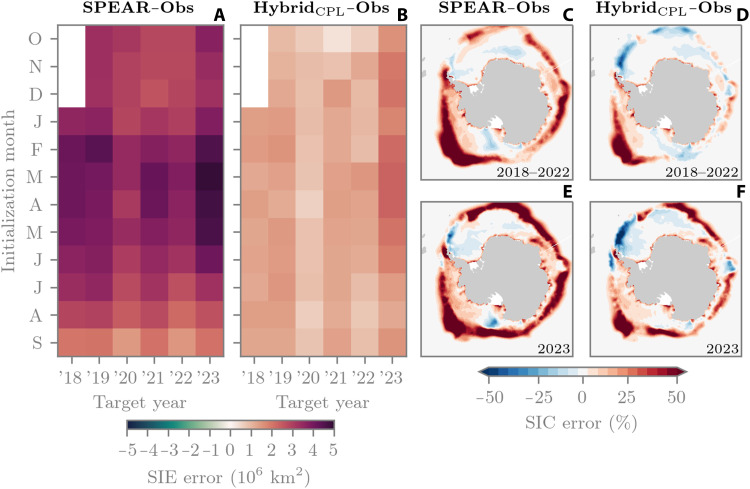
September Antarctic sea ice prediction error. (**A** and **B**) Pan-Antarctic September sea ice extent (SIE) error for each year between 2018 and 2023 for SPEAR and Hybrid_CPL_, respectively. (**C** and **D**) Mean September SIC error for March- to May-initialized reforecasts, for SPEAR and Hybrid_CPL_, respectively. (**E** and **F**) Same as [(C) and (D)] but for 2023. All errors are relative to NSIDC observations.

In Fig. 6, we show the September Antarctic sea ice prediction skill for both SPEAR and Hybrid_CPL_. In terms of pan-Antarctic sea ice extent, SPEAR has a positive September extent bias for all initialization months and years (Fig. 6A), where the bias grows steadily with increasing lead time, up to approximately February. The largest September 2023 forecast errors occur for initialization dates March–May, with an average extent bias of 4.77 million km^2^. Meanwhile, the average 2018–2022 extent bias for March to May forecasts is 3.80 million km^2^, an error increase of 0.97 million km^2^ from 2018–2022 to 2023. The September sea ice extent bias for Hybrid_CPL_ is then systematically lower than SPEAR for all initialization months and years (Fig. 6B). For years 2018–2022, the forecast error does not grow with lead time at the same rate as SPEAR. For example, the difference in sea ice extent bias for February-initialized forecasts versus September-initialized is only 0.08 million km^2^, while for SPEAR, it is 2.05 million km^2^. For March- to May-initialized forecasts in 2023, Hybrid_CPL_ shows an increase of 1.12 million km^2^ in forecast error compared to 2018–2022, from 1.21 to 2.33 million km^2^. While the magnitude of this error increase is relatively similar for Hybrid_CPL_ and SPEAR (1.12 versus 0.97, respectively), the absolute error for March to May forecasts with Hybrid_CPL_ is still >2× lower than SPEAR.

Figure 6 (C and D) shows the average September SIC error for March- to May-initialized forecasts between 2018 and 2022 for SPEAR and Hybrid_CPL_, respectively. This shows that the hybrid model is removing a significant amount of error along the ice edge and is removing some of the large SIC bias in the Ross and Amundsen seas. Comparing these figures to September 2023 (Fig. 6, E and F), we see that SPEAR has larger ice edge errors than 2018–2022, particularly in the Weddell and Ross seas, as well as the Pacific sector. SPEAR also has a localized negative SIC error in the Ross Sea ice pack. Hybrid_CPL_ shows increased ice edge errors relative to its 2018–2022 counterpart. The Hybrid_CPL_ error pattern generally resembles a muted version of the SPEAR error pattern, except for the Weddell Sea, where the errors are exacerbated, and the Ross Sea, where the negative error in SPEAR is no longer present.

This September Antarctic case study demonstrates a successful example of how hybrid models can systematically improve seasonal sea ice forecasts. However, because of the error increase between 2018–2022 and 2023 being roughly consistent for SPEAR and Hybrid_CPL_, we cannot confidently say here that Hybrid_CPL_ is better equipped to capture extreme events. Despite this, the systematic bias improvements from Hybrid_CPL_ suggest an improvement in the “quality” of our ensemble forecast system, where quality can be quantified in terms of the ratio of the ensemble mean forecast error (RMSE) to the spread (1σ) in the ensemble, the so-called spread-skill metric (*64*). This ratio should be approximately equal to 1 for a well-behaved model, while for SPEAR, it is, on average, equal to 10 for September Antarctic sea ice forecasts (see fig. S6). While the average ratio is considerably improved for Hybrid_CPL_ at 3.4, the model is still considerably underdispersive, meaning that it may still struggle to capture extreme events within its forecast ensemble; note that this spread is not the same as the spread of the DA ensemble, which generated the ML training data (see Materials and Methods for a detailed description of the differences between the forecast and DA ensemble and a discussion on the suitability of the Kalman filter for our sea ice DA workflow). Nevertheless, the improvements in sea ice mean state suggest that Hybrid_CPL_ can potentially improve the representation of coupled sea ice processes in seasonal forecasts, such as Southern Ocean net primary productivity (*65*) or surface air temperature (*66*). This goes beyond the scope of our present study, although it will be investigated in future work. Last, we note that Antarctic sea ice also experienced record low February sea ice conditions in 2023 (*67*). Given the issues surrounding Hybrid_CPL_ generalization in Antarctic summer, we do not provide a detailed analysis of this event. However, we include fig. S7 to confirm that the February Antarctic sea ice extent prediction error of Hybrid_CPL_ is higher than SPEAR for nearly all initialization dates.

## DISCUSSION

This study introduced a hybrid modeling framework that uses ML to bias correct global sea ice conditions during a set of 1-year fully coupled forecast experiments with the GFDL SPEAR climate model. The ML models in this study were trained to predict SIC DA increments using only information from local model state variables, yielding a state-dependent representation of the sea ice model errors. We have paid particular attention to how training ML models on DA increments generated from reanalysis-forced versus nudged configurations of SPEAR are able to generalize to the fully coupled free-running SPEAR model. We referred to the two resultant hybrid models from these training configurations as Hybrid_IO_ and Hybrid_CPL_, respectively.

Reforecast experiments initialized between 2018 and 2023 show that Hybrid_CPL_ outperforms SPEAR in the Arctic for all target months other than October and November, for which there are only marginal degradations in pan-Arctic RMSE of SIC (<1%). Meanwhile, Hybrid_IO_ shows systematic degradations relative to SPEAR for target months May to November (~4.5% increase in SIC RMSE), which is due to out-of-sample behavior originating from ML input variables, particularly SSS. In the Antarctic, Hybrid_IO_ also systematically degrades SPEAR forecasts between July and January (~10% increase in RMSE). This is due to a combination of out-of-sample behavior and coupled feedbacks between the ML model and physical processes within SPEAR. For one, the mean Antarctic DA increments show different spatial patterns between the reanalysis-forced and nudged fully coupled model, highlighting that these two model configurations have different sea ice biases. Therefore, learning increments in the ice-ocean model does not generalize to the fully coupled model. Furthermore, the Antarctic reforecasts with Hybrid_IO_ trigger a sequence of coupled feedbacks, whereby the ML model first creates negative SIC and thickness anomalies relative to SPEAR. This then increases ocean vertical mixing, which brings more heat to the surface and further exacerbates the negative volume anomaly. These processes considerably affect the sea ice mean state in Hybrid_IO_, with a sea ice wintertime maximum occurring 1 month earlier than under SPEAR and summertime conditions that are ice free. Conversely, the Hybrid_CPL_ configuration systematically outperforms SPEAR between May and December, reducing the September Antarctic forecast bias by more than a factor of 2. While our relatively short validation period has not allowed us to confidently assess whether Hybrid_CPL_ is more skillful at predicting sea ice anomalies, we hypothesize that an improved mean state will inherently allow the forecast ensemble to capture a more realistic range of events, as seen in other bias correction studies (*32*). Nevertheless, an extension of the present methodology could be to train on anomaly increments, which has shown success at improving the representation of large-scale atmospheric modes of variability (*48*).

The improved online generalization with Hybrid_CPL_ underscores a central takeaway from our study that exposing ML models to coupled ice-atmosphere-ocean processes is essential for robust online performance in free-running coupled model simulations. Our framework therefore provides a promising step toward improving operational numerical predictions with ML. However, achieving this goal first requires attention of some key considerations:

1) Hybrid_CPL_’s forecast degradations in Antarctic summer. We showed that these degradations are likely originating from coupled ocean model biases, which were not present in the training data due to the DA simulation containing SST nudging. While online generalization could potentially be improved by generating the sea ice DA increments in a free-running configuration of SPEAR, we have endeavored to remain consistent with past studies (*44*, *45*), which constrained the sea ice in this way to target intrinsic sea ice model physics errors. Directly targeting sea ice model physics errors allows for flexibility in terms of future model development, whereby the learned errors can be potentially attributed to specific deficiencies within preexisting parameterization schemes (*68*). In any case, we have shown that applying SST nudging on top of our ML-based bias correction in these 1-year reforecast experiments considerably improves online generalization in Antarctic summer. Therefore, future work will involve running weakly coupled DA experiments where assimilation is performed in both the sea ice and ocean components. This will provide a consistent set of ocean and sea ice increments with which to train ML models and apply together during subsequent reforecast experiments. It is then reasonable to assume that learning DA or nudging increments in the atmosphere, ocean, and sea ice together would provide a complete picture of the model’s systematic errors and is likely the most promising path forward for reducing coupled model biases with ML.

2) The computational cost and considerations for integration into operational systems. The ML model used in this study is lightweight and independent of any external libraries or specific compilers, therefore making it adaptable to any large-scale sea ice model. One crucial aspect of the architecture is that it is a local model, making predictions of the sea ice increment at each grid point using a halo of four grid points on all sides. This halo size is identical to what each processor carries when integrating the sea ice model during SPEAR simulations; thus, we avoid carrying extra data or needing to perform expensive gather operations to do ML inference. Furthermore, our proposed ML model has ~100,000 weights (see table S1), which is considerably smaller than the typical hundreds of millions of weights used in networks for applications such as climate model emulation (*69*). These decisions enable fast inference on central processing unit (CPU) hardware and ultimately mean that Hybrid_CPL_ only suffers a 0.3% performance slowdown compared to SPEAR.

3) Long-term stability and generalization. While we have showcased our ML-based bias correction framework in 1-year reforecasts here, the methodology also has potential for climate-timescale integrations. Achieving this requires further development of the methodology toward a conservative implementation of the corrections. At present, the sea ice increments are applied to the SIC state at every thermodynamic timestep by simply adding or removing sea ice within a given grid cell. In reality, these updates should also make changes to the heat, water mass, and salt content of the ocean mixed layer. Conserving heat poses substantial challenges and warrants investigation. One past study showed that a conservative ocean temperature tendency adjustment approach can be achieved by ensuring that the global integral of the temperature corrections equals zero (*32*). This is likely insufficient for our sea ice case, where we often need to make a net change to the sea ice state. However, our ML framework could be updated to conserve water mass and salt by computing an appropriate surface heat flux (q-flux) that would create the necessary SIC change predicted by the ML model, an approach that has been proposed for sea ice nudging during polar amplification model intercomparison project simulations (*70*). Conserving the water mass budget would be crucial for understanding how such an ML scheme affects large-scale overturning circulation patterns in the ocean, for example. Beyond conservation, the ML model will also need to generalize to warmer climates if used in future projection experiments. While this will be the subject of future work, we hypothesize that our current approach may already be robust for this purpose. By virtue of being a local model and the fact that SIC has a lower bound of zero, our training data contain an abundance of examples of the climate conditions that facilitate a locally ice-free state (e.g., SSTs above the freezing point). Therefore, we may expect that under future projection experiments, our ML framework is well equipped to handle transitions to an ice-free Arctic or Antarctic.

## MATERIALS AND METHODS

### The GFDL SPEAR model

The SPEAR is a fully coupled ice-atmosphere-ocean-land model (*58*). There are two configurations of SPEAR that are routinely run at GFDL for climate simulations and seasonal predictions: SPEAR_LO_ and SPEAR_MED_. These two configurations differ only in the horizontal resolution of their atmospheric and land components, at 1° (SPEAR_LO_) and 0.5° (SPEARMED_MED_), respectively. Otherwise, both configurations contain 33 vertical levels in the atmosphere, 75 vertical levels in the ocean, with the atmosphere, land, ocean, and sea ice based on AM4.0, LM4.0, Modular Ocean Model version 6 (MOM6), and Sea Ice Simulator version 2 (SIS2), respectively (*71*–*73*). The ocean and sea ice components are configured to a nominal 1° horizontal resolution in both SPEAR_LO_ and SPEAR_MED_. Although SPEAR_MED_ generally outperforms SPEAR_LO_ in terms of seasonal Antarctic sea ice forecasts (*74*), our study focuses on the relative improvements of a given climate model’s sea ice forecasts through our hybrid ML scheme. We therefore opt for SPEAR_LO_ (hereafter SPEAR) given its computational advantage.

### Generating the training data

For details of the reanalysis-forced ice-ocean simulation used to train the ML model for Hybrid_IO_, we refer the reader to studies (*44*, *45*). The model state variables and DA increments that are used to train the ML model for Hybrid_CPL_ are generated from a SPEAR 30-member large ensemble simulation spanning 1982–2017. The initial conditions for this simulation are from a perturbed physics spin-up run off of a SPEAR large ensemble historical simulation spanning 1851–2010. Specifically, we rerun the historical large ensemble simulation between 1 January 1968 and 1 January 1979 but with perturbed sea ice physics parameters for each ensemble member [see (*44*) for details of these perturbations]. Then, from 1 January 1979 to 1 January 1982, the 3D atmospheric temperature, winds, and humidity fields are nudged to the National Oceanic and Atmospheric Administration (NOAA) climate forecast system reanalysis (CFSR) (*75*) at a 6-hour e-folding timescale for temperature and winds and 24-hour e-folding timescale for humidity. From 1 January 1982 to 1 January 2018, we then nudge SSTs toward version 2.0 of the NOAA optimum interpolation SST (OISST) product (*76*) at a piston velocity of 4 meters per day, which corresponds to a timescale of 12.5 days for a 50-m mixed layer. We also nudge the atmosphere to CFSR as before and assimilate passive microwave SIC observations from NSIDC (*61*) into SIS2 using the ensemble adjustment Kalman filter (EAKF) (*77*). It should be noted that sea ice–covered grid points within the raw OISST data are assigned a fixed value of −1.8°C. During nudging, we then replace OISST values of −1.8°C with the salinity-dependent freezing point of sea water (*T*_f_) at each timestep, based on the model’s SSS and the model’s empirical freezing-point equation *T*_f_ = −0.054 SSS. Without this change, the SST nudging can trigger spurious ice-growth feedbacks in regions of fresh water such as the East Siberian and Laptev seas, which have freezing points that are warmer than −1.8°C. We also note here that SIS2 has a five-category subgrid SIT distribution, where the aggregate, or observable, SIC is a diagnostic equal to the sum of the concentration in each category. Providing observations are available, and sea ice DA is performed every 5 days over the course of the 36-year simulation, where DA first estimates the error in the model’s aggregate SIC and then, through the ensemble covariance between the model’s aggregate and category SIC, estimates errors in each category SIC. From this simulation, we then compute the 5-day mean of all model state variables, providing 2619 pairs of model state variables (inputs) and DA increments (outputs) to train the ML model.

### ML model architecture and training

The ML framework proposed in (*44*) uses a convolutional neural network (CNN) to map model state variables and their tendencies to the aggregate SIC increment from DA (the sum of the increments in each subgrid thickness category). The input variables for this CNN are SIC, SST, zonal, and meridional components of ice velocities, SIT, SWn, ice-surface skin temperature, SSS, and lastly a land-sea mask (17 inputs in total). The predicted increment from this CNN is then passed to an artificial neural network (ANN), along with state variables and tendencies corresponding to the subgrid category SIC fields and a land-sea mask, to predict the SIC DA increments of each category. In (*45*), this ML architecture was then used to bias correct ice-ocean simulations every 5 days across a 5-year simulation. While this approach systematically reduced global sea ice biases, it left egregious sawtooth-type imprints of the 5-day corrections in the resultant simulation. In fig. S8, we show that increasing the frequency of the ML corrections to 2 days in this same ice-ocean configuration (and linearly scaling the predicted increments by two-fifths), leads to poor performance. This is due to out-of-sample issues related to the model state tendencies. Therefore, by removing the tendencies from the list of inputs and retraining the networks, we achieve stable online performance at 1-day implementation frequency (fig. S8C). This 1-day implementation subsequently removes all correction imprints. We therefore use these same subsets of inputs for both Hybrid_IO_ and Hybrid_CPL_ in our present study. Specifically, the CNN uses nine inputs and the ANN uses seven inputs. Figure S9 shows a schematic of this model architecture, where the yellow squares in the CNN represent 3 by 3 convolution kernels used in all layers, and the purple squares in the ANN are the local operations occurring at each grid cell (the same as a CNN with a 1 by 1 kernel). Therefore, given four convolution operations, the ML model requires a 9 by 9 stencil to make local predictions. To ensure that data boundaries are appropriate for this 9 by 9 stencil, we pad the CNN input data during offline training with four grid points on all sides. This padding follows zonal periodicity, zero padding along the southern boundary (Antarctic continent), and symmetric padding across the Arctic bipolar fold [see (*44*) for more details].

The CNN and ANN are initially trained offline using all available training data between 1982 and 2017. Both Hybrid_CPL_ and Hybrid_IO_ follow an identical offline training procedure, except that the input and output training data for Hybrid_CPL_ are generated from a nudged configuration of SPEAR, while for Hybrid_IO_, they are generated from a reanalysis-forced configuration of SPEAR. The specific details of the network architecture and hyperparameters used during offline training are summarized in table S1. Note that the hyperparameters were selected by a grid search approach, where each particular set of hyperparameters was evaluated using a fivefold cross-validation approach to guarding against overfitting. A fivefold cross-validation means that, for each hyperparameter test, the model was trained five times, where, each time, the training data were split into different 80 to 20 training and validation chunks, respectively. These chunks were temporally contiguous to avoid data leakage associated with temporal autocorrelation within the data.

Following offline training, both Hybrid_IO_ and Hybrid_CPL_ ML models were fine-tuned according to the procedure of (*45*). This fine-tuning is designed to improve offline-to-online generalization of ML models and involves running a new simulation across the 1982–2017 training period, where sequential corrections from the offline-trained ML model and DA are applied to the SIC state every 5 days (see fig. S10). Following this simulation, the sum of the instantaneous ML and DA increments provides a new training dataset with which to fine-tune the offline-trained ML model. This fine-tuning uses the same model hyperparameters and architecture as detailed in table S1, except the training is only run for five epochs. This procedure can be run iteratively until convergence. In this study, two iterations of fine-tuning are used for Hybrid_IO_, while one iteration is used for Hybrid_CPL_ due to computational expense. Note that the performance of Hybrid_IO_ in coupled seasonal forecasts is not improved by more iterations of fine-tuning, as it has never been exposed to coupled ice-atmosphere-ocean feedbacks. Meanwhile, we may expect the performance of Hybrid_CPL_ to improve with more iterations of fine tuning.

### ML model implementation

The 1-day implementation in fig. S8C is achieved by performing offline updates to the model restart files in Python, which has a ~ 440% slowdown cost associated with pausing and restarting the model every day. To address this issue, we implement the ML models directly into the SIS2 source code and apply the corrections to SPEAR reforecasts at the sea ice thermodynamic timestep (30 min). The CNN and ANN architectures are relatively simple, consisting only of 2D convolution operations, local weighted sums, and rectified linear unit functions. We therefore also code these directly into Fortran, rather than relying on a Fortran-Python wrapper such as FTorch to do the inference (*78*).

While developing this approach, we initially encountered generalization issues related to the fact that the CNN has been trained on 5-day-mean input fields, which smooths out features including the diurnal cycle and sharp gradients associated with sub–5-daily variability, features that are prevalent in SWn, ice velocities, and surface skin temperature instantaneous fields (see fig. S11). We address this issue through a pragmatic solution of gathering the network inputs over the first day of the simulation to compute a daily mean. With these daily-mean fields, we then do inference with the ML model at 00:00 UTC and apply this predicted correction to the category SIC states at every timestep over the course of the proceeding day (while also accumulating the network inputs again for the next daily-mean computation). Note that we also scale the predicted increment by ^1^/_240_ to account for a 30-min thermodynamic timestep. This procedure then continues for the length of the simulation. Through this configuration, the network receives the same input fields as the offline restart approach, although it now spreads the corrections across each timestep. It is also worth highlighting that, during the simulation, each processor by default carries a halo of four data points on all sides, which is exactly the halo needed for our CNN. Therefore, we simply use the MOM6 internal padding routine to populate these halo points before online CNN inference. Through these intentional architecture and implementation choices, the hybrid approach maintains roughly equivalent throughput as the free-running SPEAR model (0.3% slowdown), even when doing ML inference on CPU. Last, in the case where sea ice is added to a grid cell that was previously ice free, we assign this new ice a salinity of 5 practical salinity units, a temperature of −2°C, and a thickness of 0.05, 0.2, 0.5, 0.9, 2.0 m for subgrid categories 1 to 5, respectively.

### Reforecast initialization procedure

The initial conditions are identical for the SPEAR and Hybrid reforecasts and are based on a series of ocean and sea ice DA experiments. For the ocean, initial conditions come from a 30-member SPEAR ocean DA simulation spanning 1990–2023, within which NOAA OISST data, Argo temperature and salinity floats, expendable bathythermograph data, and tropical moorings are assimilated daily using the EAKF (*32*). The sea ice, atmosphere, and land initial conditions for both SPEAR and Hybrid reforecasts correspond to simply extending the 1982–2017 sea ice DA simulation that was used to generate the ML training data from January 2018 to December 2023.

The SPEAR and Hybrid reforecasts in this study are configured as 15-member ensemble experiments that run for 1 year. This corresponds to combining the first 15 members of the atmosphere, land, and sea ice initial conditions from the sea ice DA experiment with the first 15 members of the ocean DA experiment. We note that a 15-member forecast ensemble is smaller than the 30-member DA ensemble (see section below on how these ensembles are configured). However, past literature has shown that the uncertainty on seasonal-to-interannual Arctic sea ice prediction errors approximately converge for ensemble sizes greater than 10 (*79*). Therefore, given that our results focus on ensemble-mean statistics, we expect that we would see very little difference between a 15-member and 30-member forecast ensemble.

Last, the reforecasts also include an “ocean tendency adjustment” approach, which applies a climatology correction to the 3D ocean temperature and salinity fields on month of the year (*32*). This approach has been shown to reduce ocean model bias in climate simulations with SPEAR and also improve the seasonal prediction skill of El Niño Southern Oscillation.

### Differences between the forecast and DA ensembles

In Results, on extreme events, we mentioned that the forecast ensemble for both SPEAR and Hybrid_CPL_ is underdispersive. An underdispersive ensemble would be concerning for the EAKF, as the model would become overconfident, leading to very small or zero updates when assimilating observations. However, we note in this section that the 15-member forecast ensembles of SPEAR and Hybrid models are distinct from the 30-member ensemble used for DA. The forecast ensemble uses constant sea ice physics parameters but has initial condition spread in each model component. Meanwhile, the DA ensemble achieves spread by perturbing sea ice physics parameters, where the mean of the perturbed values are centered on the values used by the forecast ensemble. We therefore expect smaller spread in the forecast ensemble than the sea ice DA ensemble. Furthermore, past studies (*34*) found that when assimilating SIC observations at the grid cell level, the spread in the model’s aggregate SIC can decrease but the model critically retains spread in the subgrid category SIC states (i.e., the states that are actually being updated during DA). We may therefore see lower spread in integrated metrics like pan-Antarctic extent (e.g, fig. S6); however, this will not necessarily translate to reduced category SIC spread at the grid scale.

Last, we acknowledge that our forecast model contains systematic model bias, which is not theoretically optimal for Kalman filter applications that assume zero-mean Gaussian error distributions. However, we do still consider it useful for the purpose of reducing initial condition errors (*34*) and learning systematic model error (as per this present study). Last, given that SIS2 has a prognostic SIT distribution ice thickness distribution (ITD), we rely on the EAKF for its ability to update the category SIC terms through the model’s own covariance structure between aggregate and category SIC. If we were instead to rely on a simpler approach like nudging, then we would need to develop ad hoc assumptions about how to update the ITD based on aggregate SIC observations.
