# Supplementary material for: Advancing global sea ice prediction capabilities using a fully coupled climate model with integrated machine learning
Source: Sci Adv. 2026 Jan 1;12(1):eady8957. doi: 10.1126/sciadv.ady8957 (PMC12757041; doi:10.1126/sciadv.ady8957)
Supplement: Supplementary file 1 — Table S1 Figs. S1 to S11 [file sciadv.ady8957_sm.pdf]

Science Advances
AAAS

Supplementary Materials for

Advancing global sea ice prediction capabilities using a fully coupled climate model with integrated machine learning

William Gregory *et al.*

Corresponding author: William Gregory, wg4031@princeton.edu

Sci. Adv. **12**, eady8957 (2026)
DOI: 10.1126/sciadv.ady8957

This PDF file includes:

Table S1
Figs. S1 to S11

Table S1: Details of the ML architectures used during training. Table adapted from (*44*) under the CC-BY 4.0 license (https://creativecommons.org/licenses/by/4.0/) to reflect the network properties used in this present study.

	**CNN**	**ANN**
**Inputs**	SIC, SST, SIU, SIV, SIT, SW, TS, SSS, land-sea mask	ΔSIC^{CNN}, SICN, land-sea mask
**Outputs**	ΔSIC	ΔSICN
**Size of input data set**	$2619 \times 9 \times 328 \times 368$	$2619 \times 7 \times 320 \times 360$
**Size of output data set**	$2619 \times 1 \times 320 \times 360$	$2619 \times 5 \times 320 \times 360$
**Normalization**	Inputs normalized to zero mean and unit variance	Inputs normalized to zero mean and unit variance
**Number of layers**	4	4
**Features per layer**	32, 64, 128, 1	32, 64, 128, 5
**Activation function(s)**	ReLU, ReLU, ReLU, Linear	ReLU, ReLU, ReLU, Linear
**Kernel size(s)**	3×3	1×1
**Kernel stride(s)**	1	1
**Bias parameters**	False	False
**Zero-padding**	None	None
**Total weights**	95,904	11,104
**Batch size**	10	10
**Optimizer**	Adam	Adam
**Learning rate**	0.001	0.001
**Weight decay**	1×10^{-7}	1×10^{-7}
**Epochs**	125	125
**Seed**	711	711

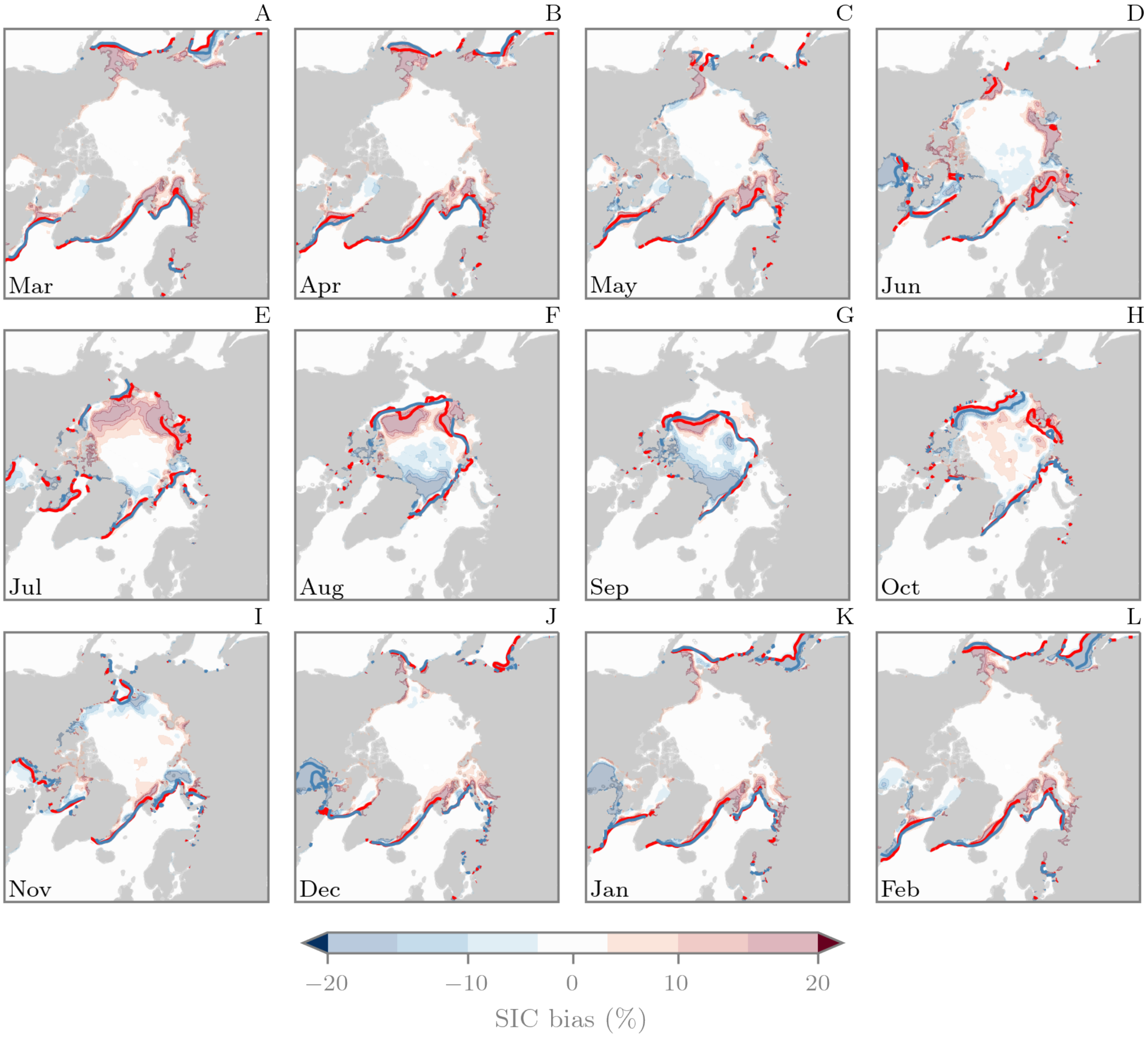

Figure S1: **March-initialized SPEAR reforecast bias, 2018–2024.** (**A–L**) March–February monthly-mean sea ice concentration (SIC) bias for SPEAR reforecasts, relative to NSIDC observations, and the associated monthly-mean sea ice edge contour for SPEAR (blue) and observations (red).

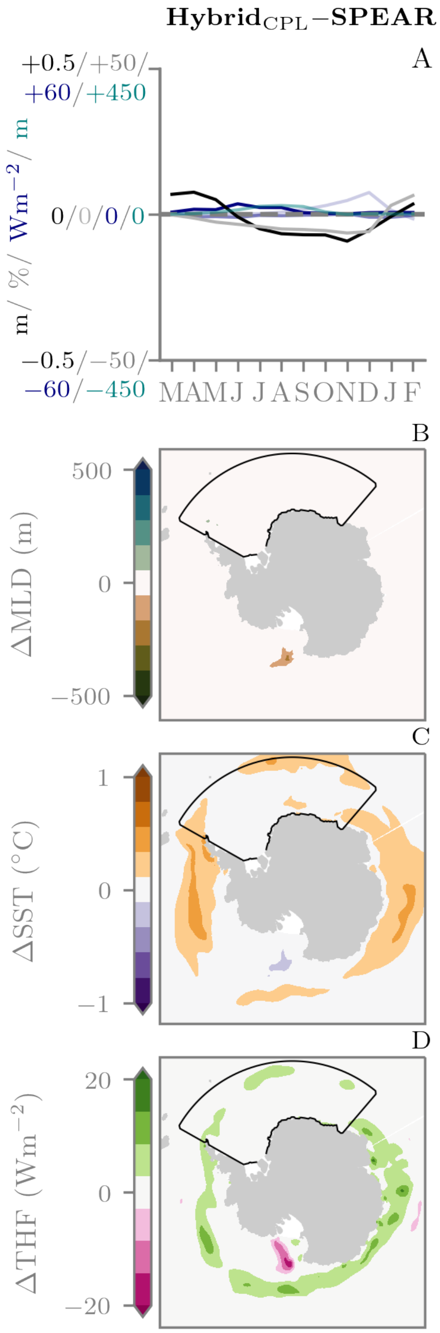

Figure S2: **March-initialized Hybrid$_{\mathrm{CPL}}$–SPEAR anomalies, 2018–2024.** Same as Fig. 4, but for Hybrid$_{\mathrm{CPL}}$.

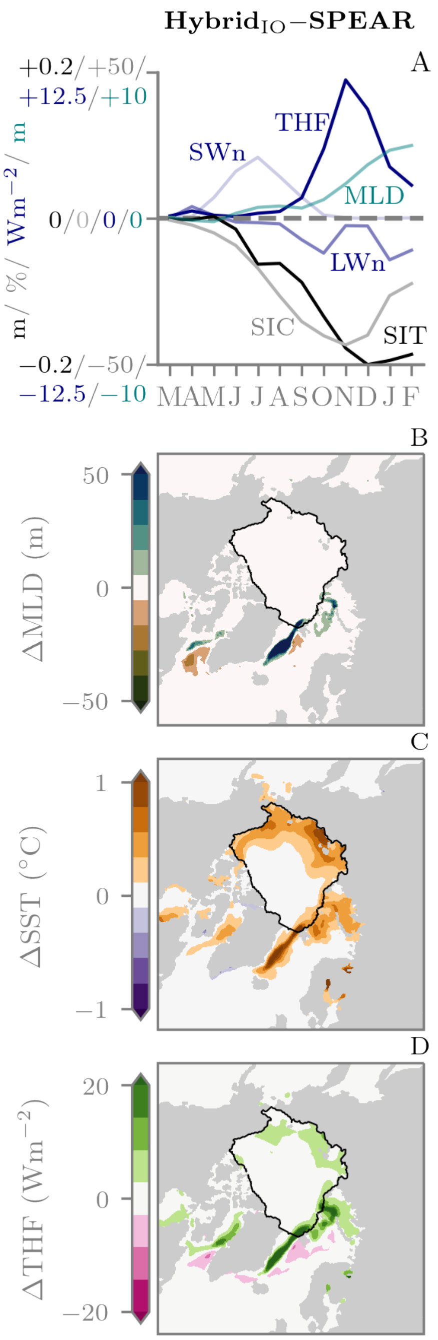

Figure S3: **March-initialized Hybrid$_{IO}$–SPEAR anomalies, 2018–2024.** (**A**) Mean Arctic basin anomalies in sea ice concentration (SIC), sea ice thickness (SIT), net shortwave radiation (SWn), net longwave radiation (LWn), turbulent heat flux (THF), and mixed-layer depth (MLD), for Hybrid$_{IO}$. THF sign convention is positive up, while LW and SW are positive down. (**B–D**). Average Hybrid$_{IO}$ anomalies in MLD, THF, and LWn across the 1-year reforecasts. Contour shows region of anomalies in (**A**).

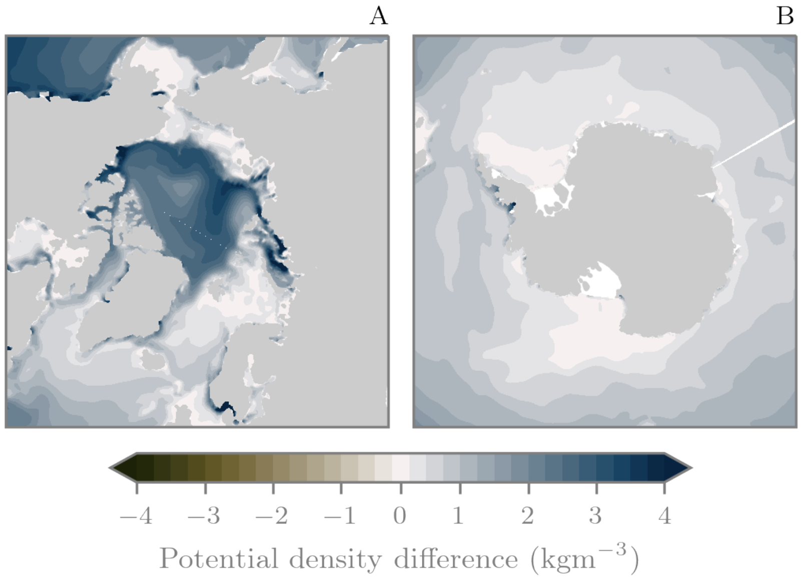

Figure S4: **SPEAR annual-mean ocean stratification indicator, 2018–2024.** (**A**) An indicator of Arctic ocean stratification defined as the difference in ocean potential density at 500 metres minus ocean potential density at surface. Values close to 0 indicate a well-mixed surface ocean, while larger values indicate a stratified surface ocean. (**B**) Same as (**A**) but for the Antarctic.

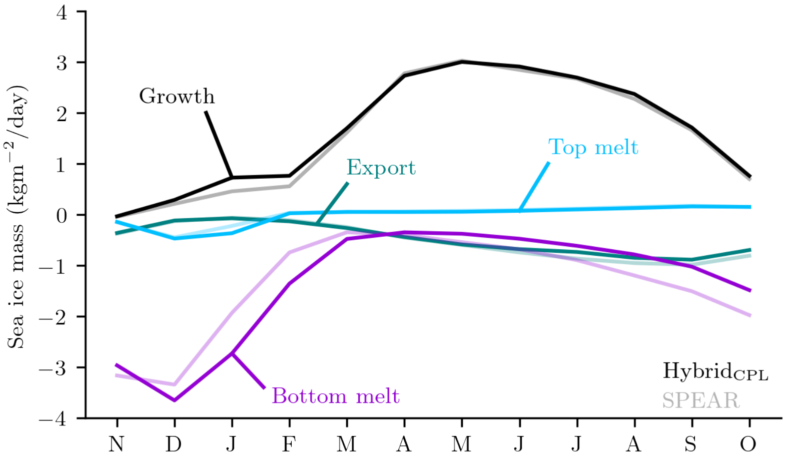

Figure S5: **Pan-Antarctic sea ice mass budget tendencies, 2018–2024.** The annual cycle of mass budget tendencies for SPEAR (transparent curves) and Hybrid$_{CPL}$ (solid curves), decomposed into growth, melt, and transport (export) terms. Generated from November-initialized reforecasts.

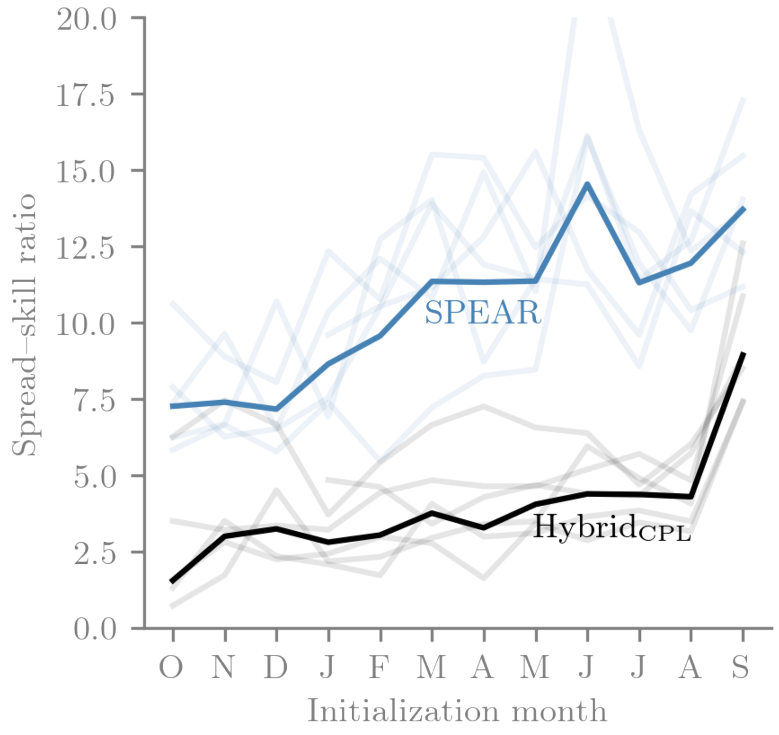

Figure S6: **Spread–skill ratio for pan-Antarctic September sea ice extent forecasts.** Transparent lines are for individual forecast years between 2018–2023, while the opaque lines are the mean across all years. Forecast lead time increases from right to left on x-axis.

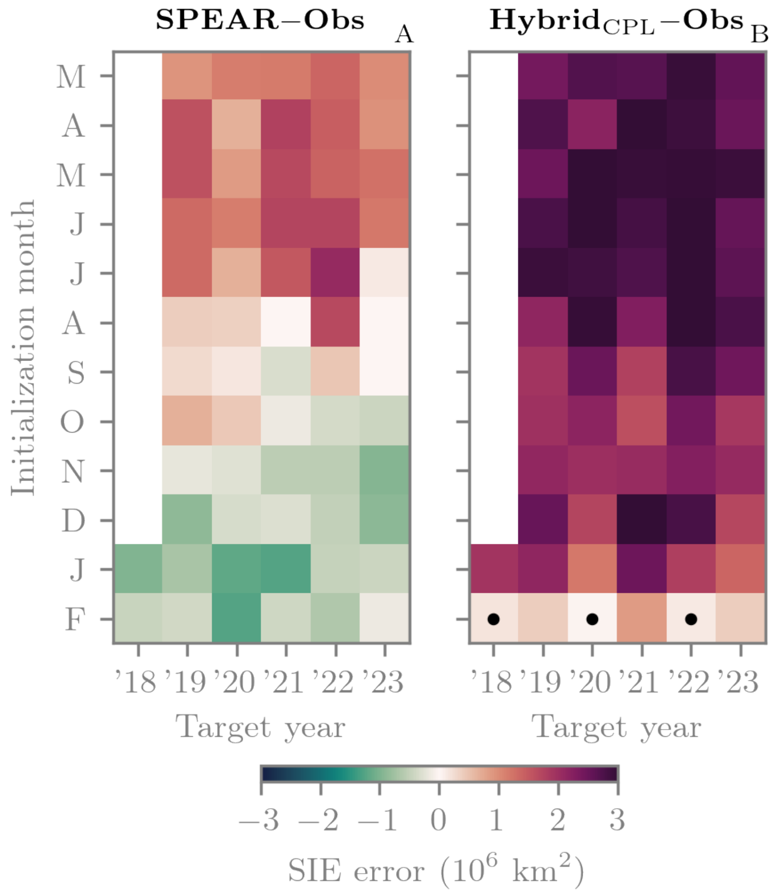

Figure S7: **February Antarctic sea ice extent prediction error.** (**A**,**B**) Pan-Antarctic February sea ice extent (SIE) error for each year between 2018–2023, for SPEAR and Hybrid$_{CPL}$, respectively. The black scatter points show where Hybrid$_{CPL}$ has a lower error than SPEAR. Errors are relative to NSIDC observations.

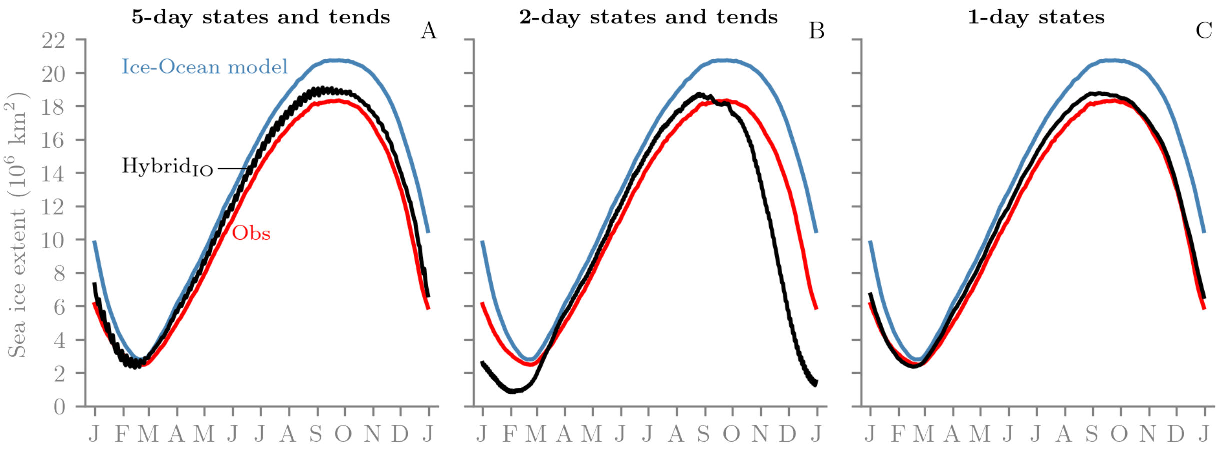

Figure S8: **Different ML implementation frequencies in ice-ocean simulations, 2018–2022.** (**A**) Mean pan-Antarctic sea ice extent for a reanalysis-forced ice-ocean model (blue), observations (red), and an ice-ocean simulation which performs ML-based bias correction every 5 days via offline updates to model restarts (black). (**B**) Using the same ML model as in (**A**) but doing ML corrections every 2 days. (**C**) Drop model tendencies from ML inputs and implement every 1 day.

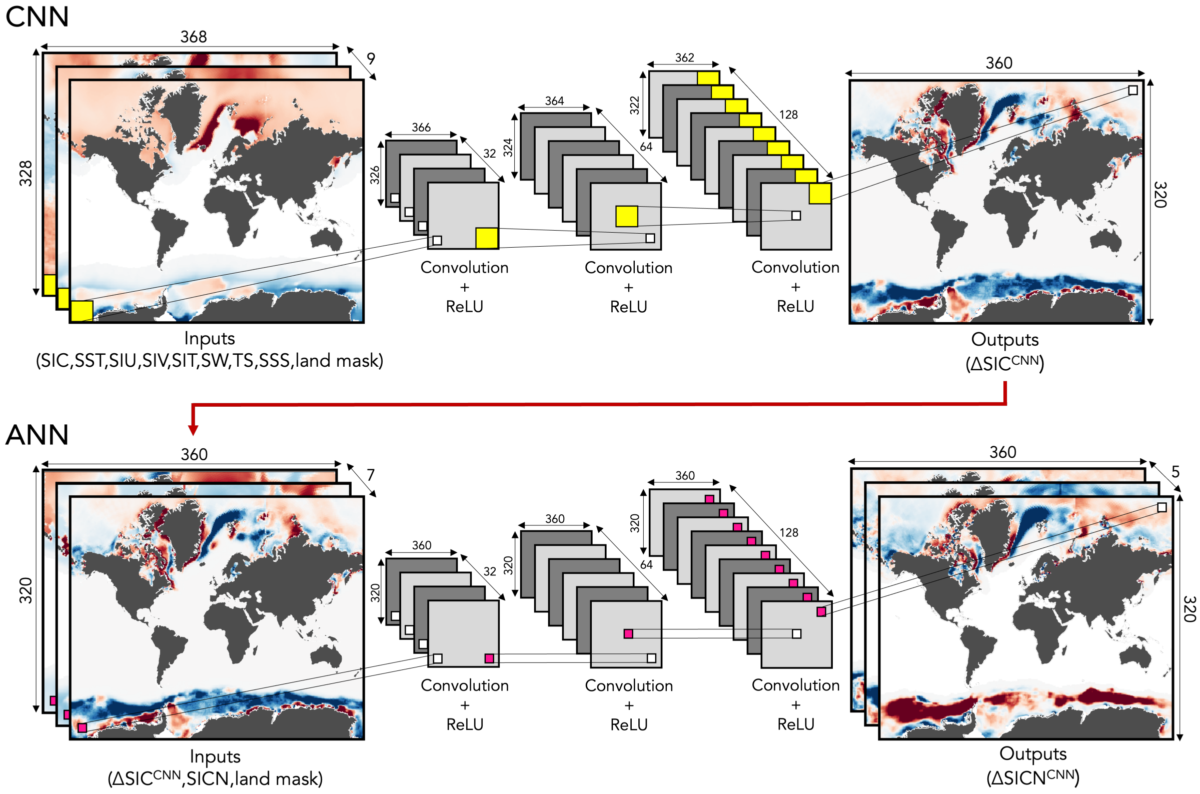

Figure S9: **The machine learning architecture used to predict sea ice concentration increments.** See Materials and Methods text for detailed description. SIC = aggregate sea ice concentration, SST = sea-surface temperature, SIU = zonal ice velocity, SIV = meridional ice velocity, SIT = sea ice thickness, SW = net shortwave radiation, TS = ice-surface skin temperature, SSS = sea-surface salinity, ΔSIC = aggregate SIC increment, SICN = category SIC, ΔSICN = category SIC increment. Figure is adapted from (*44*) under the CC-BY 4.0 license (https://creativecommons.org/licenses/by/4.0/) to reflect the network properties used in this present study.

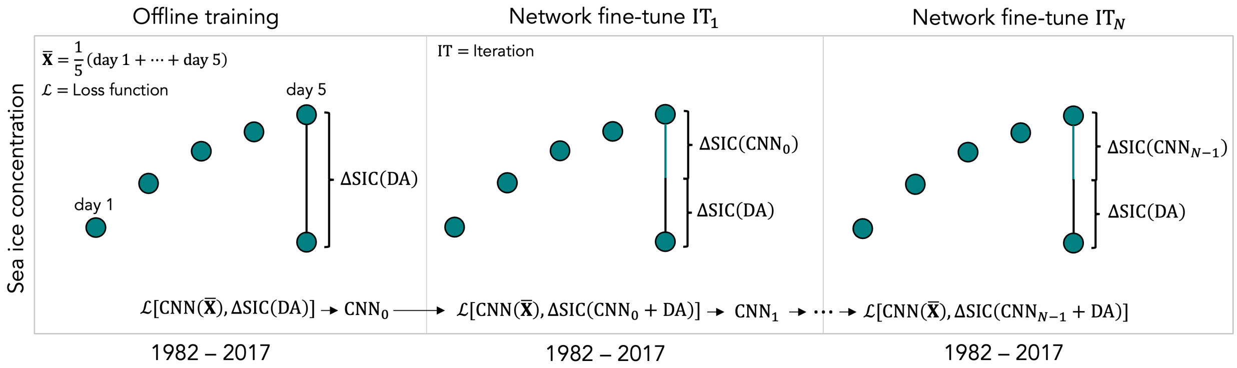

Figure S10: **Fine-tuning of machine learning models with DA.** See Materials and Methods text for detailed description. Each scatter point represents a daily-mean model state variable. Therefore, DA and/or ML updates are performed every 5 days, where the ML model is trained on the mean of the model state variables over the 5 days. Figure is adapted from (*45*) under the CC-BY 4.0 license (https://creativecommons.org/licenses/by/4.0/) to emphasize the data augmentation procedure.

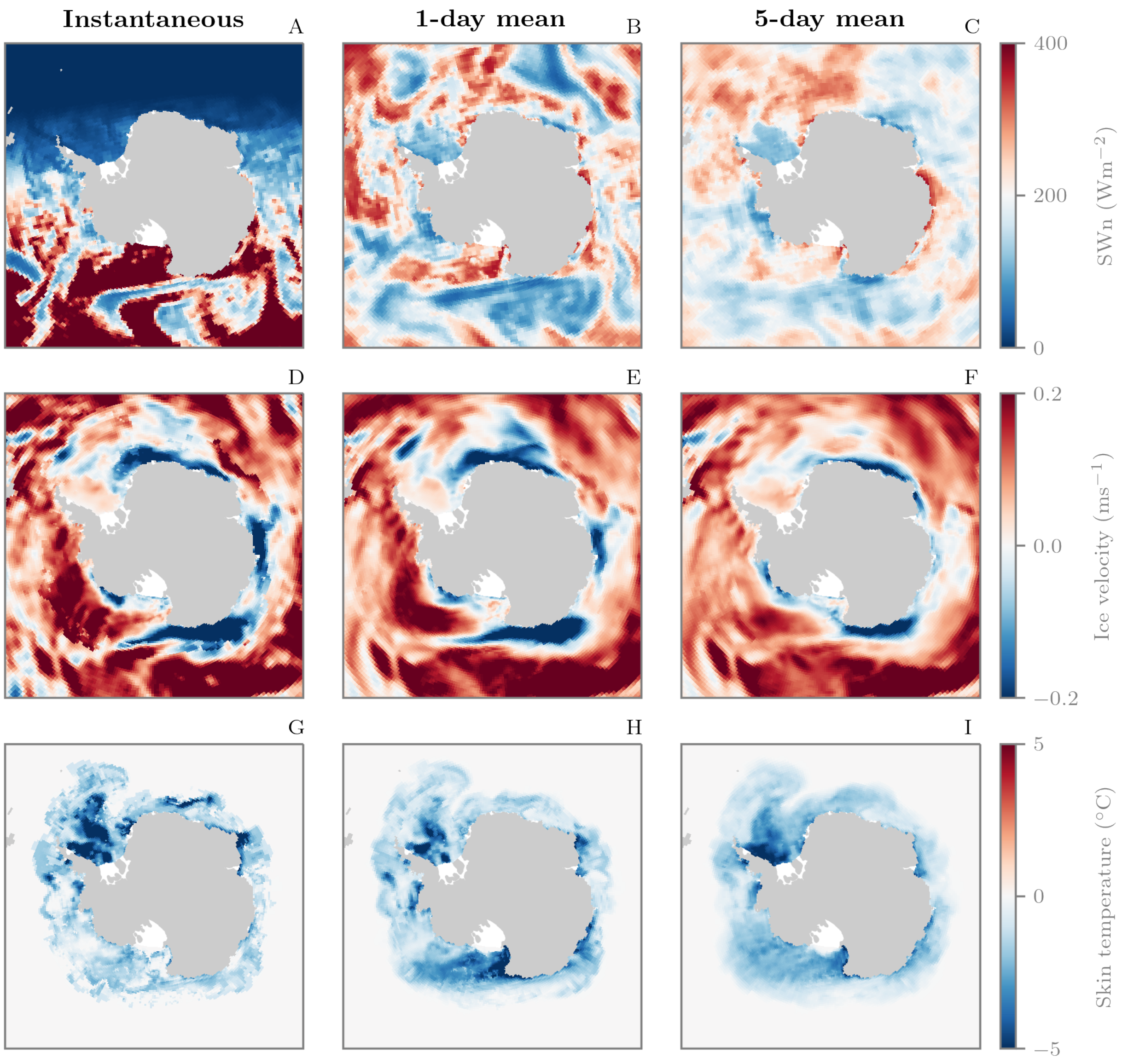

Figure S11: **Difference in ML inputs across temporal averaging windows.** (**A**) Instantaneous net shortwave (SWn) on Januay 1 2018 at 00:30 UTC, (**B**) the 1-day mean SWn from January 1 2018 00:00 UTC to January 2 2018 00:00 UTC, (**C**) the 5-day mean SWn from January 1 2018 00:00 UTC to January 6 2018 00:00 UTC. (**D–F**) Same as (**A–C**) but for zonal ice velocity. (**G–I**) Same as (**A–C**) but for ice-surface skin temperature. Notice features like the diurnal cycle in (**A**) or grid artifacts in (**D**) and (**G**). All ML models in this study are trained on 5-day mean state variables.
